# Reproducibility of planar ^123^I-meta-iodobenzylguanidine (MIBG) myocardial scintigraphy in patients with heart failure

**DOI:** 10.1007/s00259-012-2180-2

**Published:** 2012-07-13

**Authors:** Caroline E. Veltman, Mark J. Boogers, Joris E. Meinardi, Imad Al Younis, Petra Dibbets-Schneider, Ernst E. Van der Wall, Jeroen J. Bax, Arthur J. H. A. Scholte

**Affiliations:** 1 Department of Cardiology, Leiden University Medical Center, Albinusdreef 2, 2333 ZA Leiden, The Netherlands; 2 The Interuniversity Cardiology Institute of the Netherlands (ICIN), Utrecht, The Netherlands; 3 Department of Nuclear Medicine, Leiden University Medical Center, Leiden, The Netherlands

**Keywords:** Heart failure, Planar MIBG myocardial scintigraphy, Delayed heart to mediastinum (H/M) ratio, Region of interest (ROI), Reproducibility

## Abstract

**Purpose:**

Despite its high prognostic value, widespread clinical implementation of ^123^I-meta-iodobenzylguanidine (MIBG) myocardial scintigraphy is hampered by a lack of validation and standardization. The purpose of this study was to assess the reliability of planar ^123^I-MIBG myocardial scintigraphy in patients with heart failure (HF).

**Methods:**

Planar myocardial MIBG images of 70 HF patients were analysed by two experienced and one inexperienced observer. The reproducibility of early and delayed heart-to-mediastinum (H/M) ratios, as well as washout rate (WR) calculated by two different methods, was assessed using the intraclass correlation coefficient (ICC) and the Bland-Altman analysis. In addition, a subanalysis in patients with a very low H/M ratio (delayed H/M ratio <1.4) was performed. The delayed H/M ratio was also assessed using fixed-size oval and circular cardiac regions of interest (ROI).

**Results:**

Intra- and interobserver analyses and experienced versus inexperienced observer analysis showed excellent agreement for the measured early and delayed H/M ratios and WR on planar ^123^I-MIBG images (the ICCs for the delayed H/M ratios were 0.98, 0.96 and 0.90, respectively). In addition, the WR without background correction resulted in higher reliability than the WR with background correction (the interobserver Bland-Altman 95 % limits of agreement were −2.50 to 2.16 and −10.10 to 10.14, respectively). Furthermore, the delayed H/M ratio measurements remained reliable in a subgroup of patients with a very low delayed H/M ratio (ICC 0.93 for the inter-observer analysis). Moreover, a fixed-size cardiac ROI could be used for the assessment of delayed H/M ratios, with good reliability of the measurement.

**Conclusion:**

The present study showed a high reliability of planar ^123^I-MIBG myocardial scintigraphy in HF patients, confirming that MIBG myocardial scintigraphy can be implemented easily for clinical risk stratification in HF.

## Introduction

The ageing of the population and the improved medical treatment of cardiac patients has led to an increased prevalence of heart failure (HF) [1]. Despite the therapeutic innovations, the mortality rate of patients with HF remains high with an estimated 5-year mortality rate of 54 % in men and 40 % in women [2].

Increased myocardial sympathetic activity is a prominent feature of HF by which the failing heart tries to compensate for the reduced cardiac output [3]. In the chronic state of HF these compensatory mechanisms become deleterious, causing myocardial hypertrophy and fibrosis, leading to cardiac remodelling and restructuring [4]. At the cellular level, the increased sympathetic activity causes increased neuronal release of norepinephrine (NE), that leads to a significant reduction in presynaptic NE uptake due to posttranscriptional downregulation of the cardiac NE transporter [5]. The decrease in the NE reuptake mechanism can be assessed noninvasively by radionuclide imaging with the ^123^I-labelled NE analogue meta-iodobenzylguanidine (MIBG) [6]. MIBG is taken up into the presynaptic cardiac sympathetic nerves by the NE uptake-1 transporter, and the amount of MIBG retention over several hours after administration reflects neuronal integrity [7]. The most commonly used quantitative measurements of myocardial MIBG uptake are the calculated heart-to-mediastinum (H/M) ratio and washout ratio (WR) determined from planar MIBG images. A low delayed H/M ratio has been shown to be an independent predictor of ventricular tachyarrhythmia [8, 9], appropriate ICD therapy [10] and sudden cardiac death [11] in HF patients. In addition, an increased myocardial washout has been associated with an adverse prognosis [12]. Subsequently, planar MIBG myocardial scintigraphy has been demonstrated to have strong prognostic value [13, 14].

Despite the proven prognostic value of MIBG imaging in patients with HF, there are still several limitations that prevent this technique from being implemented as a clinical management tool in patients with HF [15]. Although almost all reports include the H/M ratio and WR as the measure of myocardial uptake, the methods used to obtain these parameters show substantial variation [16]. This variation can be caused by the influence of collimator choice, acquisition time and duration, and the location and size of the cardiac and mediastinal regions of interest (ROIs) [17–19]. MIBG uptake can be very low, particularly in patients with HF, hampering the assessment of the cardiac ROI on planar MIBG images. Therefore, the level of experience in drawing the cardiac ROI on the planar MIBG images might influence the measured H/M ratio and WR. Moreover, because of this difficulty in determining the correct contours of the cardiac ROI in HF patients, the assessment of the location and size of the cardiac ROI might affect the measured H/M ratio and WR more in patients with HF as compared to healthy subjects. Furthermore, different methods are used to calculate the WR from early and delayed planar MIBG images [16].

To our knowledge there are no studies that have compared the use of these different mathematical methods for the assessment of WR. Therefore, the effects of the method on the clinical interpretation of WR remain unclear, and it is uncertain which method would provide the more accurate and reproducible estimates. Consequently, the reproducibility of the clinically important parameters of planar MIBG myocardial scintigraphy remains unidentified in HF patients. Therefore, the purpose of this study was to assess the reproducibility of the H/M ratio and WR in HF patients. We evaluated the influence of two different methods to assess the WR, the effect of cardiac ROI size and position on the assessment of the H/M ratio, as well as the impact of the level of postprocessing experience on the reproducibility of planar MIBG images.

## Materials and methods

### Study population

Patient data and MIBG myocardial scintigraphy images collected as part of a previously reported clinical study were utilized in the present analysis [10]. The study population consisted of New York Heart Association (NYHA) functional class III/IV HF patients with an impaired left ventricle ejection fraction (LVEF) of ≤35 % who were clinically referred for MIBG myocardial scintigraphy. All patients were treated according to the ACC/AHA guidelines for the diagnosis and management of HF [20], receiving optimal pharmacological and revascularization therapy. From the previously described patient population a random sample of 70 HF patients was selected for the analysis of the current study. The MIBG images of the selected patients were reanalysed to evaluate the reproducibility of the H/M ratio on the planar MIBG myocardial images.

### MIBG data acquisition

Patient medication that could have influenced MIBG myocardial uptake, predominantly antihypertensive drugs and tricyclic antidepressants, were withdrawn. Patients were pretreated with 120 mg of sodium iodide to block uptake of free ^123^I by the thyroid gland. Sodium iodide was given orally 1 h before intravenous administration of 185 MBq of MIBG (AdreView; GE Healthcare, Princeton, NJ). MIBG planar imaging was performed with the patient in the supine position. A 10-min planar anterior thoracic image was acquired (256 × 256 matrix) 10–15 min after tracer administration. Planar imaging was repeated approximately 4 h after tracer administration (delayed images). All camera heads were equipped with low-energy high-resolution collimators and all images were acquired with a 15 % energy window centred at the 159 keV photopeak of ^123^I.

### Planar MIBG image analysis

**Fig. 1:**
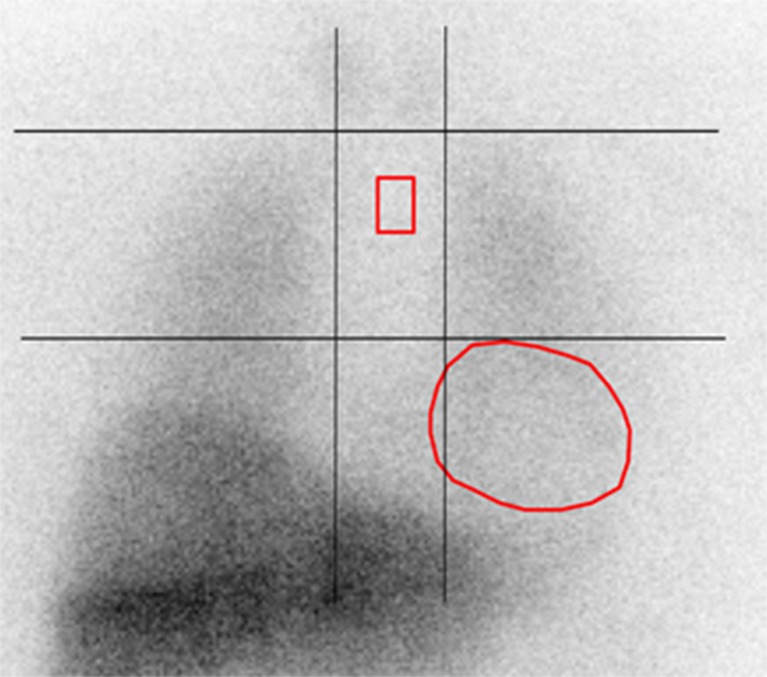
The mediastinal ROI and the manually drawn polygonal cardiac ROI on a planar MIBG image. The rectangular mediastinal ROI (size 13 × 20 pixels) was placed in the upper half of the “anatomical landmark square” formed by the lung apexes (upper square border), the upper cardiac border (lower square border) and the medial contours of the lungs (medial square borders). The manually drawn polygonal cardiac ROI was placed over the myocardium including the left ventricular cavity

H/M ratios were calculated from planar MIBG images using a ROI placed over the heart and over the upper mediastinum. Using dedicated postprocessing software on a Syngo-MI workstation (Siemens Medical Solutions, Malvern, PA), the cardiac ROI was assessed using a manually drawn polygonal ROI placed over the myocardium including the left ventricular cavity on the planar MIBG images (Fig. 1). The mediastinal ROI with a rectangular shape was placed on the upper half of the mediastinum and had a size of 13 × 20 pixels. The location of the mediastinal ROI was determined using the following anatomical landmarks: the lung apex as upper border, the upper cardiac border and the medial contours of the lungs (Fig. 1). The H/M ratio was computed by dividing the average number of counts within the cardiac ROI by the average number of counts within the mediastinal ROI. No background correction was performed in this study.

The WR was calculated from early and delayed planar images, using the two different methods as described in the proposal for standardization by Flotats et al. [21]. In method 1 the following formula was used: WR = (*H*_e_ − *H*_1_)/*H*_e_ × 100. In method 2 the formula with background (BKG) correction was used: WR_BKG-corrected_ = [(*H*_e_ − *M*_e_) − (*H*_l_ − *M*_l_)]/(*H*_e_ − *M*_e_) × 100. In these two formulas *H* represents the mean counts per pixel in the cardiac ROI and *M* represents the mean counts per pixel in the mediastinal ROI in the early (e) and delayed (l) planar images. In this study, time decay was not corrected for in the calculation of WR.

### Reproducibility of H/M ratio on planar MIBG imaging

To assess the reproducibility of the measured H/M ratios and WR, the intra- and interobserver agreements were tested. Intraobserver variability was assessed by a single reviewer (C.E.V.) who reviewed all planar MIBG images twice, with at least 4 weeks between the first and the second review to avoid recall bias. For the interobserver analysis two reviewers (C.E.V. and M.J.B.) independently reviewed all planar MIBG images. Both observers were experienced in the analysis of MIBG myocardial scintigraphy. The reproducibility of the delayed H/M ratio measurements between an experienced (C.E.V.) and an inexperienced observer (J.E.M.) was also assessed. The inexperienced observer was given 2 h of training in the postprocessing of planar MIBG images. During this training, the postprocessing technique and the exact location of the mediastinal ROI and cardiac ROI were explained and five example cases were evaluated.

### Subanalysis for reproducibility in patients with a very low delayed H/M ratio

Because the reproducibility of planar MIBG imaging might be different in patients with a very low delayed H/M ratio, a subanalysis was performed including only patients with a delayed H/M ratio of ≤1.4. The first measurements of one experienced observer (C.E.V.) were used to divide the study population into two groups: patients with a delayed H/M ratio of ≤1.4 were included and patients with a delayed H/M ratio of >1.4 were excluded. After selection of the patients with a very low delayed H/M ratio, the reproducibility of the delayed H/M ratio in this subpopulation was assessed by intra- and interobserver analyses.

### Image analysis using a manually drawn cardiac ROI or a fixed-size cardiac ROI

**Fig. 2:**
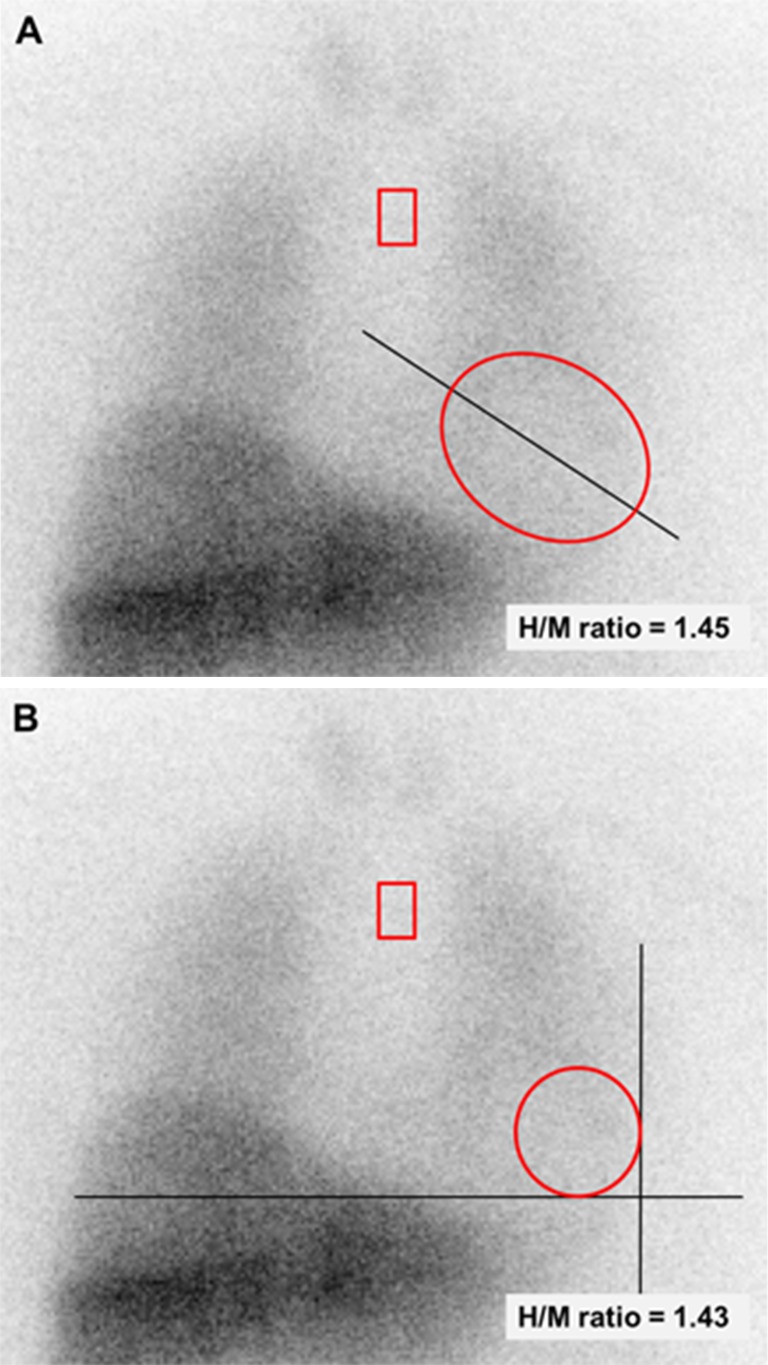
Oval and circular fixed-size cardiac ROIs on planar MIBG images. **a** The oval cardiac ROI was placed over the myocardium including the left ventricular cavity and had a size 60 × 70 pixels (approximately 85 × 100 mm). The long axis of the oval was in line with the heart axis. **b** The circular cardiac ROI had a predefined radius of 21 pixels (approximately 30 mm) and was placed at the apex of the heart, covering a part of the myocardium and left ventricular cavity. Using the oval and circular cardiac ROIs, the calculated H/M ratios were 1.45 and 1.43, respectively. In this patient the H/M ratio using the polygonal manually drawn cardiac ROI was 1.46

An additional analysis was performed to assess the reproducibility of the delayed H/M ratio using a fixed-size cardiac ROI or a manually drawn ROI. In this analysis the agreement between these two measurements was tested. Two types of fixed-size cardiac ROI were used. First, an oval cardiac ROI was placed over the myocardium including the left ventricular cavity. The size of the oval ROI was 60 × 70 pixels (approximately 85 × 100 mm) and the long axis of the oval was in line with the heart axis (Fig. 2a). The second type of fixed-size cardiac ROI tested was circular with a predefined radius of 21 pixels (approximately 30 mm). The ROI was placed at the apex of the heart, covering part of the myocardium and left ventricular cavity (Fig. 2b). The mediastinal ROI was not changed in this analysis and was determined as previously described and depicted in Fig. 1.

### Statistical analysis

All continuous variables are expressed as means ± SD. Intraclass correlation coefficients (ICC) were calculated to assess the reliability; a two-way mixed model was used with statistical significance set at 95 %. Single measures are reported for the intraobserver calculations and average measures for the interobserver calculations. For clinically relevant agreement the following criteria were used: ICC values <0.49, 0.49–0.59, 0.60–0.74 and >0.74 were considered poor, fair, good and excellent, respectively. Bland-Altman plots were used to evaluate the correlations between the measured values. The sample size was calculated using the method described by Shoukri et al. for the design of reliability studies [22]. Furthermore, the ICC was assumed to be at least 0.75. Accepting a 95 % confidence interval (CI) width of the ICC of 0.20 70 patients was found to be sufficient when measurements were performed by two independent observers. All statistical analyses were performed using the SPSS software package, version 17.0 (SPSS, Chicago, IL).

## Results

### Patient population

A total of 70 randomly selected patients (64.5 ± 8.7 years of age, 53 (76 %) men) were included in the study. The baseline characteristics of the patient population are shown in Table 1. The mean NYHA functional class was 2.6 ± 0.5, and the mean LVEF was 26 ± 7.4 % (Table 1). Of the 44 patients, 63 % had ischaemic cardiomyopathy. Medication consisted of angiotensin-converting enzyme inhibitors or angiotensin II antagonists (89 % of patients), beta-blockers (70 % of patients), lipid-lowering agents (69 % of patients) and diuretics (91 % of patients).

**Table 1:** Characteristics of the study population (*n* = 70)

Characteristic	Value
Gender (male), *n* (%)	53 (76)
Age (years, mean ± SD)	64.5 ± 8.7
Heart failure characteristics, *n* (%)	
Ischaemic cardiomyopathy	44 (63)
Nonischaemic cardiomyopathy	26 (37)
NYHA functional class (mean ± SD)	2.6 ± 0.5
LVEF (%, mean ± SD)	26 ± 7.4
Clinical cardiovascular risk factors, *n* (%)	
Diabetes	9 (13)
Hypercholesterolaemia^a^	17 (24)
Hypertension^b^	22 (31)
Family history of coronary artery disease^c^	20 (29)
Current smoking	22 (31)
Obesity (body mass index ≥30 kg/m^2^), *n* (%)	22 (31)
Medication, *n* (%)	
Angiotensin-converting enzyme inhibitor/angiotensin II antagonist	62 (89)
Beta-blocker	49 (70)
Lipid-lowering agent	48 (69)
Diuretic	64 (91)

^a^Serum total cholesterol ≥230 mg/dl and/or serum triglycerides ≥200 mg/dl or treatment with a lipid-lowering drug.

^b^Systolic blood pressure ≥140 mm Hg and/or diastolic blood pressure ≥90 mm Hg and/or the use of antihypertensive medication.

^c^Presence of coronary artery disease in a first degree family member at <55 years in men and <65 years in women

### Reproducibility of planar MIBG imaging

**Fig. 3:**
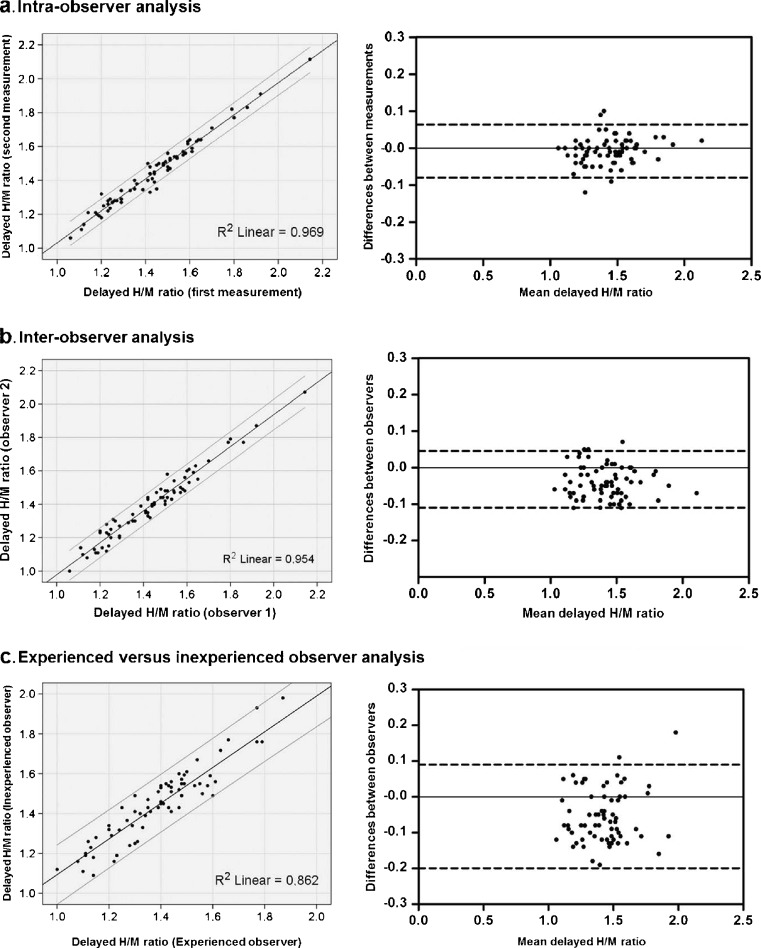
Reproducibility of the delayed H/M ratios determined on planar MIBG images: **a** intraobserver agreement (first versus second measurement), **b** interobserver agreement, **c** experienced versus inexperienced observer agreement. The mean difference (95 % limits of agreement) in the intraobserver analysis was −0.008 (−0.079 to 0.064), in the interobserver analysis was −0.040 (−0.115 to 0.046), and in the experienced versus inexperienced observer analysis was −0.053 (−0.199 to 0.094), respectively

**Fig. 4:**
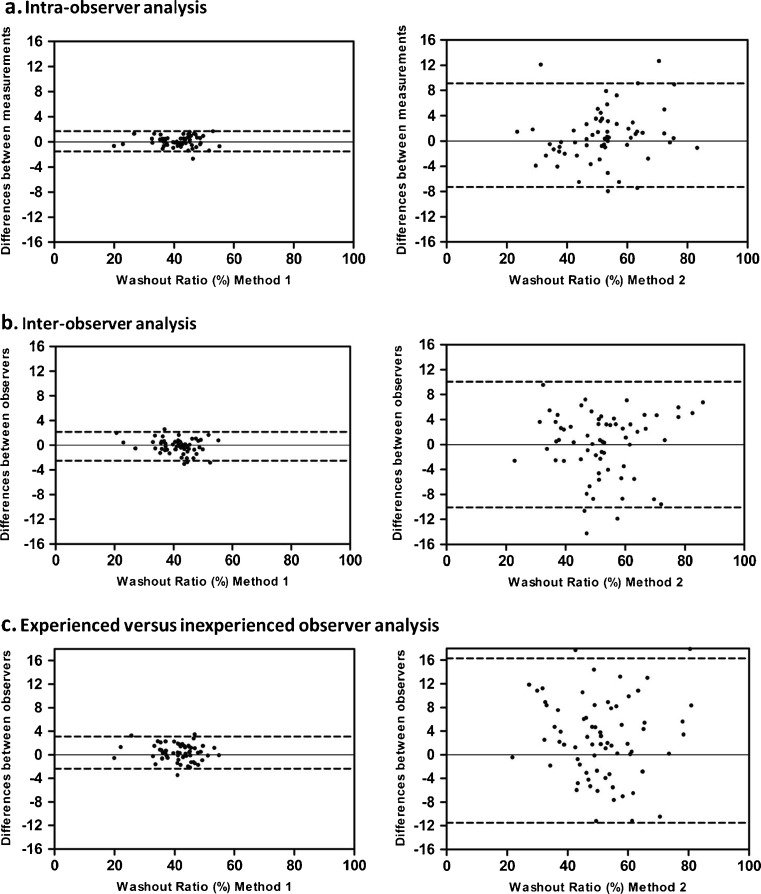
Reliability of the WR on planar MIBG images calculated using method 1 (*left panels*) and method 2 (*right panels*): **a** intraobserver agreement, **b** interobserver agreement, **c** experienced versus inexperienced observer agreement. The mean differences (95 % limits of agreement) in the intraobserver analysis were −0.096 (−1.52 to 1.71) for method 1 and −0.38 (−7.34 to 9.07) for method 2, in the interobserver analysis were −0.18 (−2.50 to 2.16) for method 1 and 0.87 (−10.10 to 10.14) for method 2, in the experienced versus inexperienced observer analysis were −0.36 (−2.39 to 3.11) for method 1 and 2.38 (−11.50 to 16.26) for method 2, respectively

The intra- and interobserver agreements, as well as the agreement between experienced and inexperienced observers for the early and delayed H/M ratio and the WR are summarized in Table 2. The interobserver analysis and the experienced versus inexperienced observer analysis showed small mean differences between the measured delayed H/M ratios (Fig. 3). WR calculated from the early and delayed planar images using method 1 was more reliable than that calculated using method 2 (Table 2). This was confirmed by the Bland-Altman analysis, which showed that WR calculated using method 2 had a wider range with larger mean differences between measurements (Fig. 4).

**Table 2:** Agreements between the measurements of the early and delayed H/M ratios and WR for the intra- and interobserver analyses and the experienced versus inexperienced observer analysis

	Early H/M ratio	Delayed H/M ratio	Washout rate (%)	
			Method 1	Method 2
Intraobserver analysis				
First measurement	1.56 ± 0.18	1.43 ± 0.20	41.5 ± 6.6	52.6 ± 13.0
Second measurement	1.56 ± 0.18	1.44 ± 0.20	41.4 ± 6.4	51.9 ± 12.5
ICC (95 % CI)	0.99 (0.98–0.99)	0.98 (0.97–0.99)	0.99 (0.98–0.99)	0.94 (0.89–0.96)
Interobserver analysis				
First observer	1.52 ± 0.20	1.40 ± 0.20	41.3 ± 6.0	52.3 ± 13.6
Second observer	1.56 ± 0.18	1.44 ± 0.20	41.4 ± 6.4	52.6 ± 13.0
ICC (95 % CI)	0.94 (0.70–0.98)	0.96 (0.76–0.98)	0.98 (0.96–0.99)	0.91 (0.85–0.94)
Experienced versus inexperienced observer analysis				
Experienced observer	1.52 ± 0.20	1.40 ± 0.20	41.3 ± 6.0	52.3 ± 13.6
Inexperienced observer	1.54 ± 0.17	1.45 ± 0.19	41.1 ± 6.5	49.5 ± 13.6
ICC (95 % CI)	0.89 (0.81–0.94)	0.90 (0.70–0.95)	0.95 (0.92–0.97)	0.81 (0.70–0.88)

### Subanalysis for the reproducibility in patients with very low delayed H/M ratio

In total, 37 patients (53 %) had a delayed H/M ratio of ≤1.4 and were selected for the subanalysis. The reproducibility of the measured delayed H/M ratio remained excellent with an ICC of 0.93 (95 % CI 0.85–0.96) and 0.93 (95 % CI 0.74–0.97), for the intra- and interobserver analyses, respectively (Table 3).

**Table 3:** Intra- and interobserver agreement for patients with a low delayed H/M ratio (≤1.4)

Analysis	Delayed H/M ratio	ICC for H/M ratio (95 % CI)
Intraobserver		
First measurement	1.28 ± 0.11	0.93 (0.85–0.96)
Second measurement	1.30 ± 0.10	
Interobserver		
First observer	1.25 ± 0.11	0.93 (0.74–0.97)
Second observer	1.28 ± 0.11	

### Fixed-size cardiac ROI

**Fig. 5:**
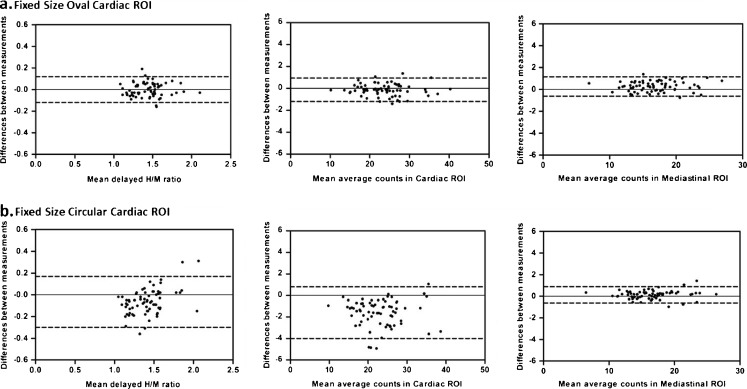
Reliability of H/M ratios measured on delayed planar MIBG images using fixed-size cardiac ROIs. Bland-Altman plots of the differences between measured H/M ratios and the mean H/M ratios (*left*), the mean average cardiac counts (*centre*), and the mean average counts in the mediastinal ROI (*right*) using the manually drawn polygonal cardiac ROI and the fixed-size cardiac ROIs. The mean difference (95 % limits of agreement) between the measured H/M ratios using the oval cardiac ROI (**a**, *left*) was −0.002 (−0.12 to 0.12), and using the circular cardiac ROI (**b**, *left*) was −0.063 (−0.30 to 0.17). This larger mean difference in mean H/M ratios was a result of the wider spread and lower average counts in the cardiac ROI (**b**, *centre*)

The H/M ratios measured using the oval cardiac ROI and the circular cardiac ROI showed excellent agreement compared to the ratios measured using a manually drawn polygonal cardiac ROI, with ICCs of 0.95 (95 % CI 0.93–0.97) and 0.86 (95 % CI 0.72–0.93), respectively (Table 4). The Bland-Altman analysis showed mean differences in the H/M ratios of −0.002 ± 0.063 for the oval cardiac ROI (Fig. 5a, left) and −0.063 ± 0.12 for the circular cardiac ROI (Fig. 5b, left), and showed good agreement between the mean counts measured in the oval cardiac ROI and in the manually drawn cardiac ROI, with 95 % limits of agreement of −1.19 to 0.95 (Fig. 5a, centre), but lower mean counts in the circular cardiac ROI than in the manually drawn cardiac ROI, with 95 % limits of agreement of −4.01 to 0.82 (Fig. 5b, centre).

**Table 4:** Agreement between the delayed H/M ratios measured in the manually drawn and fixed-size cardiac ROIs

Cardiac ROI	Mean average counts per mm^2^	Delayed H/M ratio	ICC for H/M ratio (95 % CI)	
Fixed-size oval	23.60 ± 5.67	1.44 ± 0.20	0.95 (0.93–0.97)	
Manually drawn	23.52 ± 5.64	1.43 ± 0.20		0.86 (0.72–0.93)
Fixed-size circular	22.13 ± 5.68	1.38 ± 0.20

## Discussion

The present study showed excellent reproducibility of the assessment of the most reported clinical measurements on planar MIBG images in HF patients. In particularly, when using a manually drawn polygonal cardiac ROI the highest level of agreement was observed between the measured H/M ratios and WR. However, a fixed-size cardiac ROI can also be used for the assessment of delayed H/M ratios, since high agreement was seen between the measured delayed H/M ratios obtained using a traditional manually drawn cardiac ROI and those obtained using a fixed-size cardiac ROI. In addition, for the calculation of WR, the formula using only the cardiac ROI from the early and delayed planar MIBG images (WR = (*H*_e_ − *H*_1_)/*H*_e_ × 100) provided more reliable values than the formula including background correction. Excellent agreement was also observed between the measurements of the H/M ratios and WR performed by an experienced and an inexperienced observer. A short period of training on the postprocessing technique was enough to accurately assess both the mediastinal ROI and the cardiac ROI, showing that a limited amount of experience in combination with basic anatomical knowledge is sufficient to successfully reproduce the results of an experienced observer. Subsequently, the results for the intra- and interobserver analyses could be reproduced in a subpopulation of patients with a very low H/M ratio, showing that the reproducibility remained excellent over the total range of delayed H/M ratios. These results imply that the measurements on planar MIBG myocardial scintigraphy are reliable even in patients with severe myocardial sympathetic denervation patterns.

Several studies have shown the excellent prognostic value of delayed H/M ratios and WR independent of other commonly used clinical parameters such as LVEF [12–14]. In addition, patients with decreased delayed H/M or increased myocardial MIBG washout show a higher risk of ventricular arrhythmias and sudden cardiac death [8–11]. Therefore, MIBG myocardial scintigraphy could assist in a more individualized treatment strategy for HF patients. Moreover, changes in adrenergic cardiac activity in response to pharmacological treatment in patients with HF can be tracked with the use of MIBG imaging [23–25]. However, even though multiple studies support the usefulness of this technique, MIBG myocardial scintigraphy is not yet widely clinically implemented. One factor hampering the clinical implementation of MIBG myocardial scintigraphy is the lack of standardization of the acquisition and postprocessing parameters [18]. Improved standardization of imaging protocols could contribute to increased applicability of MIBG imaging in HF patients [16].

Although the H/M ratio obtained from planar images has been used as a quantitative parameter in MIBG myocardial scintigraphy, H/M ratios may vary markedly according to the imaging equipment, especially with different types of collimator [18, 26]. However, Nakajima et al. have recently shown that standardization of H/M ratios by a heart chest calibration phantom method is feasible among different collimator types, allowing practical multicentre comparisons of H/M ratios [27]. While the effect of collimator selection on H/M ratios has been studied, similar attention should be paid to the variation caused by differences in setting the mediastinal and cardiac ROIs. Many independent reports from centres in different continents have shown that MIBG myocardial scintigraphy provides valuable prognostic information. However, among these different investigations there is no consensus on the shape, size and positioning of the ROIs for the heart and the mediastinum.

 The mediastinal ROI on planar cardiac ^123^I-MIBG images reflects nonspecific mediastinal tissue activity and is a good reference site for the qualification of the cardiac sympathetic innervation pattern, because of the small amount of scatter and the low sympathetic activity in the mediastinum [28]. Different sizes for the mediastinal ROI have been reported, ranging from 7 × 7 pixels to 9 × 9 and 40 pixels with a 128 × 128 matrix, and from 13 × 13 pixels to 20 × 20 and 100 pixels with a 256 matrix [16]. However, mostly the use of a rectangular mediastinal ROI has been reported with unspecified size [16]. Since a rectangular mediastinal ROI is recommended in the proposal for standardization by Flotats et al. [21], we chose to use a rectangular mediastinal ROI with a size of 13 × 20 pixels (with a 256 × 256 matrix) placed in the upper part of the mediastinum. A standardized shape and size of the mediastinal ROI is preferable, because small differences in size can cause variations in the measured H/M ratio [17]. Furthermore, on the determination of WR there is little consensus in the literature. Over the different prognostic studies, more than six different methods are described for the calculation of the WR from the early and delayed planar MIBG images [16]. In addition, the normal ranges and cut-off values for the WR remain unclear, while the normal values are method-dependent. Importantly, the discrepancies that arise from the use of the different mathematical methods are clinically relevant [16]. In the current analysis, the most reliable results for the calculation of the WR in HF patients were provided by calculation without background correction and without the use of ^123^I time decay correction. Therefore, this method might be the most appropriate for the calculation of WR in HF patients.

Furthermore, for the assessment of the cardiac ROI in the present study, a polygonal manually drawn cardiac ROI was used including the myocardium and the left ventricular cavity, which resulted in excellent intra- and interobserver agreement of the early and delayed H/M ratios obtained. However, a fixed-size cardiac ROI may be advantageous for future development of standardized and automatically traced cardiac ROIs. Therefore, we evaluated the reproducibility of delayed H/M ratios using two types of fixed-size cardiac ROIs. Both the oval and the circular fixed-size cardiac ROIs showed excellent correlations with the manually drawn cardiac ROI (ICC 0.95 and 0.86, respectively). However, slightly more variation was observed when the circular cardiac ROI was used. This variation could be explained by the fact that the circular ROI covers only a part of the heart. To prevent scatter from the lungs or liver, a circular ROI with a radius of 21 pixels was chosen, fitting into the apex of the myocardium without covering surrounding lung or liver tissue. However, because in most HF patients the left ventricle is enlarged, a circular cardiac ROI with a radius of 21 pixels (approximately 30 mm) often covers only a small part of the myocardium in these patients. This might influence the accuracy of the measured delayed H/M ratios in these patients. Okuda et al. used a cardiac ROI with a radius of 30 mm in the development of a semiautomated algorithm for the calculation of the H/M ratio, showing high reproducibility (ICC 0.97 in the interobserver analysis of the delayed H/M ratio) [19]. However, this study was performed in 37 patients of whom only 5 had HF. For future development of a semiautomated algorithm for the calculation of the H/M ratio in HF patients, the use of a fixed oval cardiac ROI is probably more suitable, because a larger amount of the myocardium is covered using this method.

When routine clinical application of MIBG myocardial scintigraphy is encouraged by the effort to standardize the imaging procedure, knowledge about the acquired level of experience needed to evaluate the images would be valuable. This study showed that the early and delayed H/M ratios and WR measured by an inexperienced observer were in excellent agreement with the measurements of an experienced observer. A 2-h period of training examining five example cases was enough for accurate setting of the mediastinal and cardiac ROI. This indicates that the data analysis and the interpretation of planar MIBG images are feasible. Therefore, MIBG myocardial scintigraphy could be implemented easily in the clinical work-up of HF patients. Moreover, the subanalysis performed in patients with very low delayed H/M ratios showed that the reliability remains excellent over the total range of observed myocardial sympathetic activity patterns detected on MIBG myocardial scintigraphy in HF patients.

### Limitations

The current study had several limitations. First, only the reproducibility was evaluated in this study, while the precision of planar MIBG myocardial scintigraphy also depends on the validity of the measurements performed. Second, the reproducibility of MIBG myocardial scintigraphy within subjects could not be evaluated, because MIBG myocardial scintigraphy was not repeated in time within the same patients. In the current study existing data were used of one-time MIBG myocardial scintigraphy evaluations. For the within-subject reproducibility of planar MIBG myocardial scintigraphy, a prospective study with repeated MIBG myocardial scintigraphy over time within the same patient is required. However, repeated MIBG myocardial scintigraphy within the same patient would involve an increased radiation dose. Third, we did not evaluate the influence of the size and location of the mediastinal ROI on the measured H/M ratio. However, the size of the mediastinal ROI was fixed (13 × 20 pixels) and the location was specified so that the variation in the mediastinal ROI was minimal in the current analysis (as shown in the Bland-Altman plot in Fig. 4). Still, an automatic method to set the mediastinal ROI as “default” in order to correct any variation due to the operator may be advisable. Fourth, analyses for the assessment of regional myocardial sympathetic activity on MIBG SPECT images were not performed in the current study. Instead, this study focused mainly on measurements on planar MIBG images. Further studies are needed to investigate the reliability of cardiac MIBG SPECT imaging.

### Conclusion

The present study showed excellent reproducibility of planar MIBG myocardial scintigraphy in HF patients. Moreover, for the calculation of WR, the formula without background correction showed high reliability and might therefore be preferable in HF patients. Furthermore, we observed only a small influence of the level of experience on the assessment of H/M ratios and WR, indicating that the data analysis and interpretation of planar MIBG images is feasible. In addition, the use of a fixed-size oval cardiac ROI could make the image analysis procedure even more standardized. Consequently, these results could contribute to the standardization of the data analysis and interpretation of planar MIBG images and therefore might provide extra validation for the reliability of planar MIBG myocardial scintigraphy in HF patients.
